# Optimizing Bladder Resin Transfer Molding Process to Manufacture Complex, Thin-Ply Thermoplastic Tubular Composite Structures: An Experimental Case Study

**DOI:** 10.3390/polym13234093

**Published:** 2021-11-24

**Authors:** Somen K. Bhudolia, Pavel Perrotey, Goram Gohel, Sunil C. Joshi, Pierre Gerard, Kah Fai Leong

**Affiliations:** 1 School of Mechanical and Aerospace Engineering, Nanyang Technological University, 50 Nanyang Avenue, Singapore 639798, Singapore; GORAM001@e.ntu.edu.sg (G.G.); mscjoshi@ntu.edu.sg (S.C.J.); MKFLEONG@ntu.edu.sg (K.F.L.); 2 Carbon Axis, 34 Rue Jacques de Vaucanson, 17180 Perigny, France; pperrotey@carbon-axis.com; 3 Groupement de Recherche de Lacq, Arkema Group, Route Départementale 817, 64170 Lacq, France; pierre.gerard@arkema.com

**Keywords:** thermoplastic resin, resin transfer molding, non-crimp fabrics, consolidation

## Abstract

The bladder molding process is primarily used in sporting applications but mostly with prepregs. Bladder-Assisted Resin Transfer Molding (B-RTM) presents the tremendous potential to automate and mass produce the complex hollow-composite profiles. Thin-ply, non-crimp fabrics (NCFs) provide excellent mechanical, fracture toughness, and vibration damping properties on top of the weight saving it offers to a final product. However, these fiber architectures are difficult to inject due to the resistance they provide for the polymer flow using the liquid injection process. Therefore, it is mandatory to optimize the process parameters to reduce the time for injection and simultaneously achieve better consolidation. This work presents a first, detailed, experimental case study to successfully inject a low-permeability, thin-ply, complex, thermoplastic tubular structure, and the effect of process parameters, boundary conditions, the associated manufacturing challenges, and proposed solutions are deliberated in this paper.

## 1. Introduction

The utilization of a resin transfer molding (RTM) manufacturing technique is becoming more popular in composites’ manufacturing industries [1,2,3,4]. In a RTM manufacturing technique, the polymer matrix is infused into the dry fiber preform at a certain pressure into the closed mold to impregnate the fabrics, in contrast to the traditional prepreg processing manufacturing technique [5].

Bladder-assisted resin transfer molding (B-RTM) is a manufacturing process variant specifically suited for the fabrication of hollow-composite, complex-shaped parts such as a hollow tube [4]. Hollow-composite structures are very fascinating and find tremendous applications in sailing ships (booms), wind turbine blades, pressure vessels, and in sports industries. The widely used manufacturing process for the hollow-composite profile is filament winding where the rovings are wound around the rotating mandrel to produce parts like drive shafts, tubes, and pressure vessels [6,7]. However, this process is associated with surface finishing issues, as there is no defined geometry, as well as the constraint on the fiber angle to be placed on the mandrel. Another effective process is a pultrusion process, which offers a substantially greater degree of automation for producing hollow-composite profiles [8]. However, the nature of the processing does not allow curvature within the composite structure. Although there exist some solutions such as bonding the structures together, it reduces the mechanical performance of the structure, due to existing joining lines, and there is no structural continuity. These processes are effective but do not fulfill the growing needs of the industries, such as being reliable, with reproducible parts, and making the manufacturing process automated.

**Figure 1:**
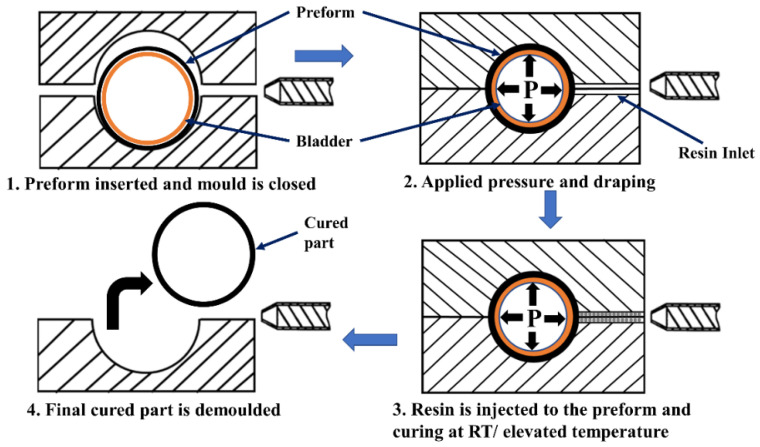
Schematic of the Bladder-assisted Resin Transfer Molding (B-RTM) process.

B-RTM is another interesting process that emerged to produce hollow-composite profiles using the Resin Transfer Molding Process with the aid of a bladder. This process is primarily used in industries with prepregs. The prepreg is wrapped around the inflatable bladder, which is consequently pressurized within a closed mold. Then, the mold is placed in the autoclave or hot press, whichever is appropriate depending on the mold geometry and the recommended cure cycle, to cure the hollow part. However, due to the sticky nature of the prepreg materials, there is a reduction in the drapability of the pre-impregnated fabrics. Although impregnated fabrics are always a solution, there is also poor reproducibility. However, B-RTM presents the tremendous potential to automate, and mass produce the complex hollow-composite profiles. The Schematic of the B-RTM process is shown in Figure 1. The major steps involve placing the preform inside the mold, pressurizing with the inflated bladder, injecting the resin, as with other liquid injection processes, and, finally, demolding the part.

There is some research carried out on investigating the B-RTM process, but it is mostly focused on woven fabrics or bi-axial braided fabrics, which have high permeability [9,10,11,12]. The B-RTM process depends on a number of factors that affect the final manufactured part such as process parameters, such as injection pressure, bladder pressure, and consolidation pressure, and the intrinsic parameters including resin viscosity and the fiber permeability [9,10,13,14,15]. Christian Schillfahrt et al. investigated the impregnation behavior of a tubular preform with respect to variable injection and bladder pressures using biaxial braided sleeving and an elastomeric silicon bladder [10]. The moldability zone for the study with Toho Tenax HTS40 carbon/corn oil was described as the one where the consolidation pressure was greater than the minimum bladder compaction pressure, and the initial bladder compaction pressure should be greater than the minimum bladder pressure required for full compaction [10]. In another study, Schillfahrt et al. presented a methodology to determine the preform compaction behavior with an undersized elastomeric bladder during the B-RTM process [11].

However, there is minimal research carried on understanding the implications of injecting the fabrics, such as low permeability, thin-ply, non-crimp fabrics (NCFs). This research work presents a first attempt to carry out a detailed experimental case study to manufacture a composite tube mimicking a section of a racket shaft using thin-ply NCFs [8,16,17,18] as a reinforcement with novel thermoplastic Elium^®^ [19,20,21,22,23,24,25,26,27,28] and thermoset epoxy resin. The details of the effective mold design and the development of injection strategy with controlled usage of process parameters are explained in detail. The effect of the process parameters governing the B-RTM process was deliberated and the final quality of the manufactured parts was checked in terms of fiber volume fraction, void content, and surface finish. This case study will serve as a guide to effectively use bladder resin transfer molding to manufacture thin- as well as thick-ply composites with thermoset and thermoplastic resin with viscosity ≤ 250 cP.

## 2. Materials and Manufacturing

### 2.1. Materials

Thin-Ply, non-crimp fabric NCCFs (0/45 bi-angle ply C-Ply™, 200 gsm) and thick-ply NCCFs (0/45 bi-angle C-Ply™, 400 gsm) from CHOMARAT were the reinforcement material used in the current research. Woven, glass fibers were procured from Polymer Technologies, Singapore, and used in the initial phase of the manufacturing optimization. C-Ply™ fabrics with 0/45 orientation were sized especially to be compatible with the respective thermoset and thermoplastic resin systems. FOE sizing was used to manufacture thermoplastic composites and Epoxy-sized fibers were used to manufacture thermoset composites.

**Figure 2:**
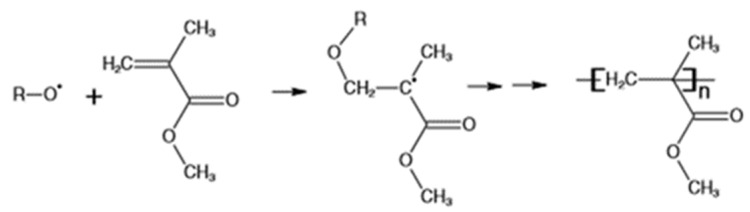
Radical polymerization of Elium matrix to form high-molecular-weight acrylic co-polymers with the addition of a benzoyl peroxide initiator [2,5,31].

Elium^®^ 150 resin from ARKEMA was used as a thermoplastic matrix system in the current investigation. This resin cured at room temperature (RT) by undergoing radical polymerization to form higher-molecular-weight acrylic co-polymers with the addition of a benzoyl peroxide initiator (refer to Figure 2) at a mixture ratio of resin to hardener by weight of 100:3 [29,30]. Epolam 5015/5015, a thermoset variant resin, was used in the current investigation. The resin was used with the hardener at a ratio of 100:30 by weight, with a curing time of 24 h at room temperature. Elium^®^ 150 resin has a viscosity of 100 cP, while the mixed viscosity of epoxy resin was 210 cP.

A slightly over-sized nylon bladder with a perimeter of 60 mm was used for inflation during the manufacturing process to provide a better compaction, whereas the inner perimeter of the final part was 56.5 mm. Table 1 shows the permeability values for thick and thin NCCFs used in current research.

### 2.2. Theoretical Background

Parameters used during the injection process play a key role to perform the complete and fast injection of the composite part [9]. However, these parameters are needed to be optimized for the selected fiber and matrix systems. Darcy’s Law oversees the resin flow through the porous medium. In the RTM process, fabric preform is deemed as a porous media and the fabric permeability depends on fiber sizing, structure, and the required volume fraction required. As the permeabilities of the fabrics varied in different directions, the resin flow through the preform is considered an anisotropic. Darcy’s Law can be written as [3],

(1) $$\bar{U}=-\frac{K}{\mu }\times \nabla P$$

where $\bar{U}$ is averaged flow velocity, μ is the viscosity of the resin, ∇P is the pressure gradient, and K is the permeability tensor of the fabric preform. Therefore, based on Darcy’s law equations, the resin flow velocity was highly dependent on the resin viscosity, preform permeability in two directions, and the pressure gradient.

#### 2.2.1. Mold Design

**Figure 3:**
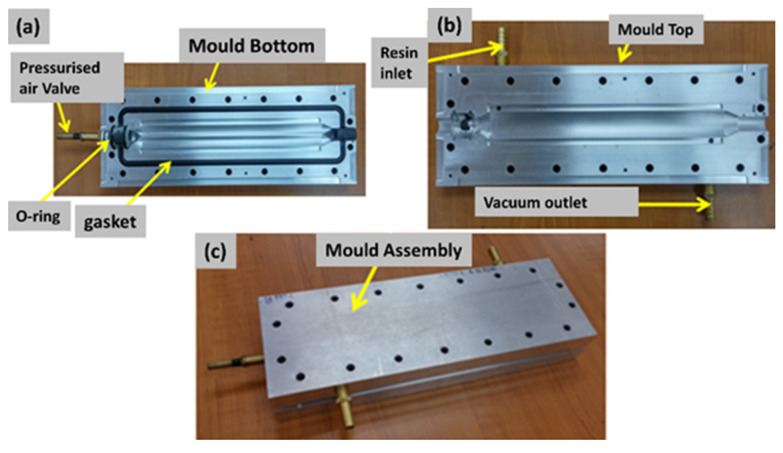
Mold design for manufacturing a tubular section (**a**) bottom mould (**b**) top mould (**c**) mould assembly.

**Figure 4:**
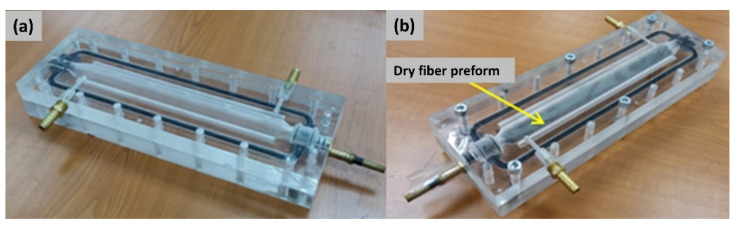
Acrylic mold prototype design for manufacturing a tubular section (**a**) mould without preform (**b**) mould with dry fabric preform.

B-RTM mold is required to be designed such that it is sealed and closed to resist pressure up to 20 bar, similarly to the RTM manufacturing process. However, using an internal bladder makes the design more complicated, as the bladder is passing through the mold, and makes the sealing more difficult. Figure 3 shows the mold used in the current research. The mold was manufactured of aluminum to reduce weight and to accelerate the heat transfer rate. The grooves were designed to insert a gasket (refer to Figure 3a) to seal the mold. The resin inlet and vacuum outlet are shown in Figure 3b, while the entire mold assembly is depicted in Figure 3c. The injection strategy was to inject the resin from one extremity of the beam and vent from the other extremity. A resin track was used to help spread the resin along the length, and the same track was added on the vacuum side to allow the excess resin to vent. It was also decided to first carry out the manufacturing trials on an acrylic prototype of the mold to visualize the resin injection and to verify if the injection strategy is working efficiently. Figure 4 shows the acrylic tubular section mold, where the cavity, as well as the resin inlet/vacuum outlet, is shown. Figure 4a shows the entire mold assembly, while Figure 4b shows the dry fiber preform placed in the mold cavity.

#### 2.2.2. Preforming, Fabrication, and Manufacturing

**Figure 5:**
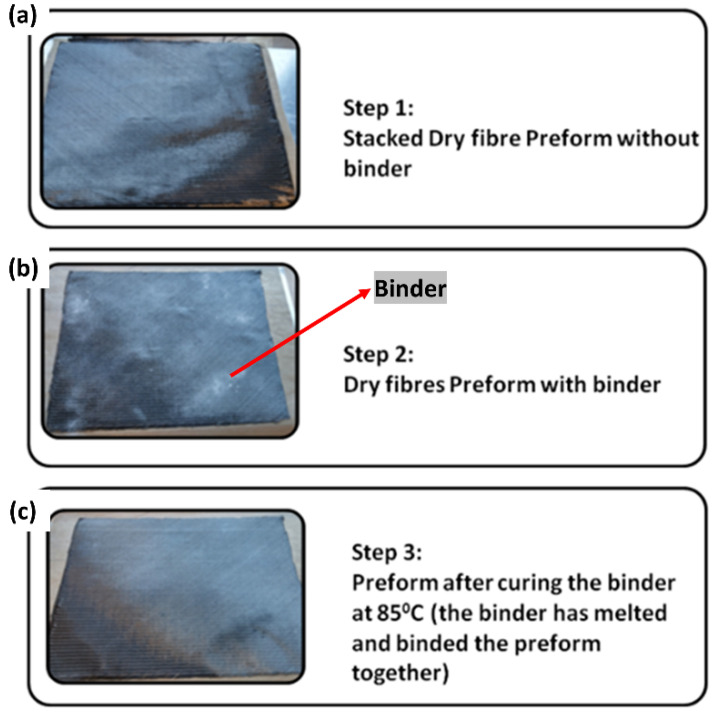
Preform binding steps for a tubular section.

At first, the dry fabric NCCF layers were stacked depending on the required layup and thickness. Then, the preform was bound with an epoxy binder based on bisphenol A. After uniformly spreading the binder on the preform, it was activated at 85 °C using the heat gun. The binder was melted and subsequently bound to the preform to wind it around the mandrel. The entire preform binding steps are shown in Figure 5. Figure 5a shows the preform without the binder sprinkled on it. Figure 5b shows the step where the binder was sprinkled on the preform. Figure 5c shows the binder was melted/activated on the preform with the heat application using the heat gun. Applying and activating the binder on the preform is significant to make a firm 3-D perform.

**Figure 6:**
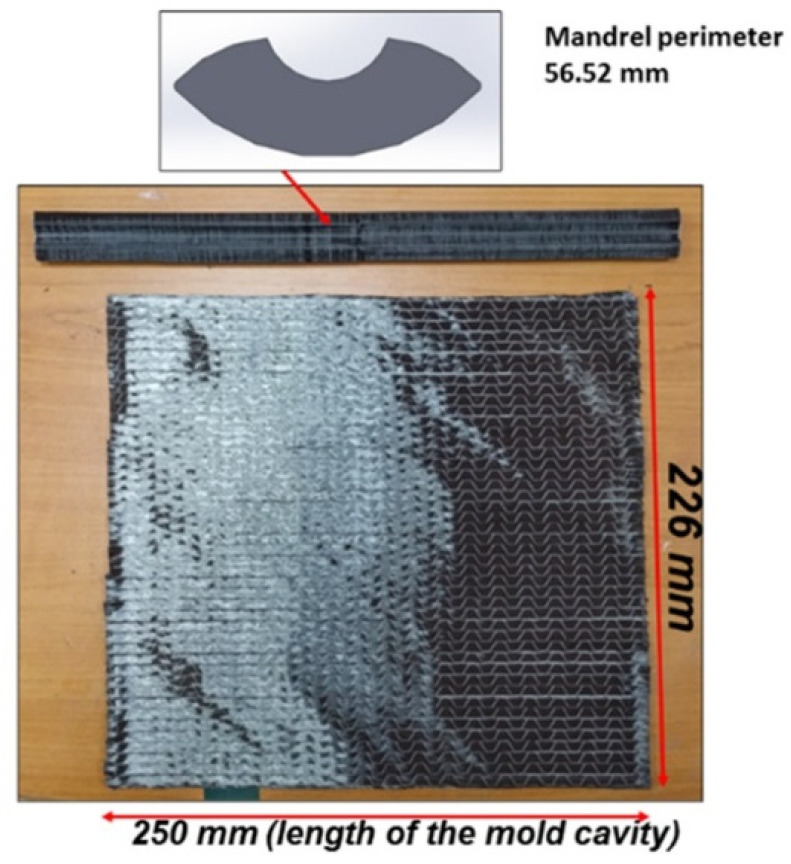
Section, mandrel, and preform dimensions.

**Figure 7:**
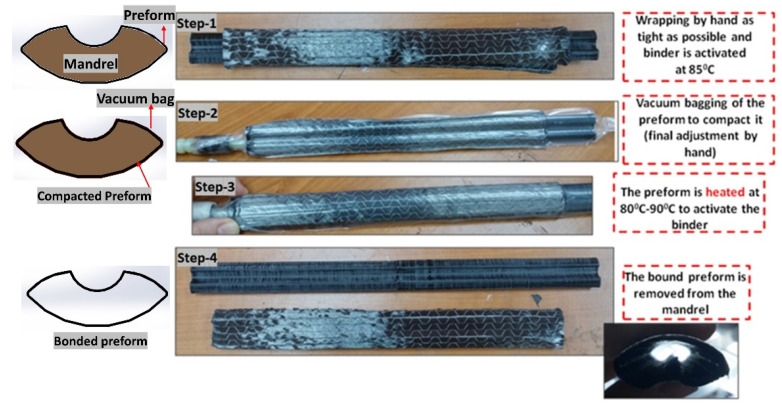
Fabrication steps to manufacture a tubular section.

**Figure 8:**
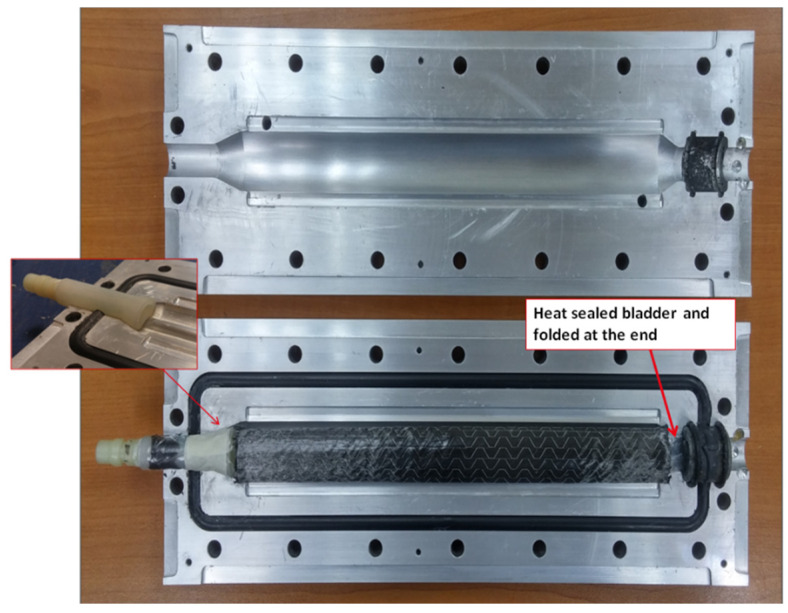
Preform positioning in the mold.

The dimensions of the target tubular section mimicking a tennis racket section, the 3-D printed mandrel, and the preform are shown in Figure 6. The preform used was 250 mm long, having the same length as the mold cavity. The width of the preform was equal to the flank length of 226 mm, which corresponded to four layers when rolled, as the perimeter of the c/s is 56.52 mm. The fabrication steps to manufacture a tubular section are shown in Figure 7. First, the preform was wrapped as tightly as possible, and the binder was activated by heating with a heat gun at 85 °C. Once the preform was wrapped around the mandrel, it was vacuum bagged for compaction. The final adjustment for net-shape preforms was carried out manually. Then, the bagged preform was heated again to reactivate the binder. Finally, the bound preform was removed from the mandrel, as shown in the last step of Figure 7. Figure 8 shows the positioning of the final, bound preform in the mold cavity. The bladder was heat-sealed and folded at the end, as shown in Figure 8.

B-RTM setup for manufacturing a tubular section is shown in Figure 8. The RTM process, utilizing a pressure pot, was chosen to inject the resin in the mold. The pressure pot system used allowed a maximum injection pressure of 5 bar. The used pressure pot system is convenient while injecting small parts as it uses pre-mixed resin in relatively small quantities compared to a dedicated RTM machine with mixing in the head. Additionally, for the fast-curing resins, as the setup uses disposable pipes, there were no risks of damaging anything as the pipes containing cured resin will be thrown away after use.

**Figure 9:**
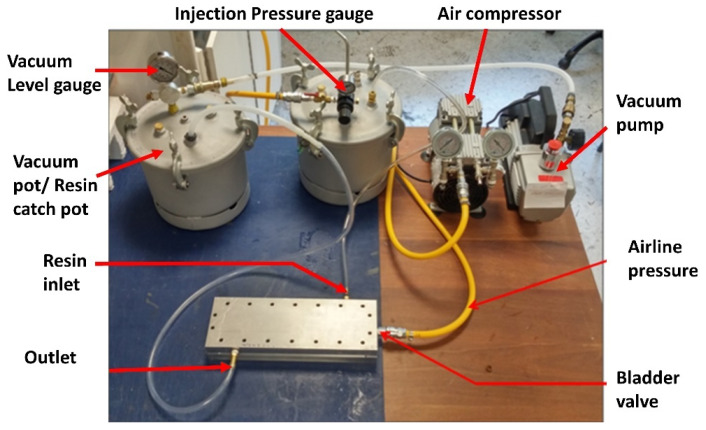
B-RTM setup for manufacturing a tubular section.

Figure 9 shows the complete test se-up for injecting a tubular section. As can be seen from Figure 9, the mold had a resin inlet, which was connected to the injection pressure pot, which, in turn, had compressed air from the air compressor. Additionally, there was a separate vacuum pot with an adjustable gauge to control the vacuum level in the outlet and a bladder valve attached to the air pressure line with a maximum capacity of 6 bar. At first, the preform was compacted with the pressurization of the inflated nylon bladder, followed by resin injection and further consolidation at higher bladder pressure to achieve a higher fiber volume fraction.

## 3. Experiments

The B-RTM process is influenced by many process parameters [9]. For the RTM process, the major process parameters that affect the impregnation, filling, quality, and fiber volume fraction of the laminates are typically (1) temperature of the mixed resin during the injection, (2) temperature of the mold, and (3) resin injection pressure or flow rate. For B-RTM, in addition to the abovementioned parameters, two more parameters arise due to the pressurization of the bladder, namely, the bladder pressure during the injection and the bladder pressure after the injection, i.e., during the curing stage [9]. There are a few studies carried out optimizing these parameters for high-permeability fabrics, such as mats, but a complete study is required to be carried out for low-permeability, Thin NCCF fabrics with novel Elium^®^ and the epoxy resin.

### 3.1. Initial Trials

**Figure 10:**
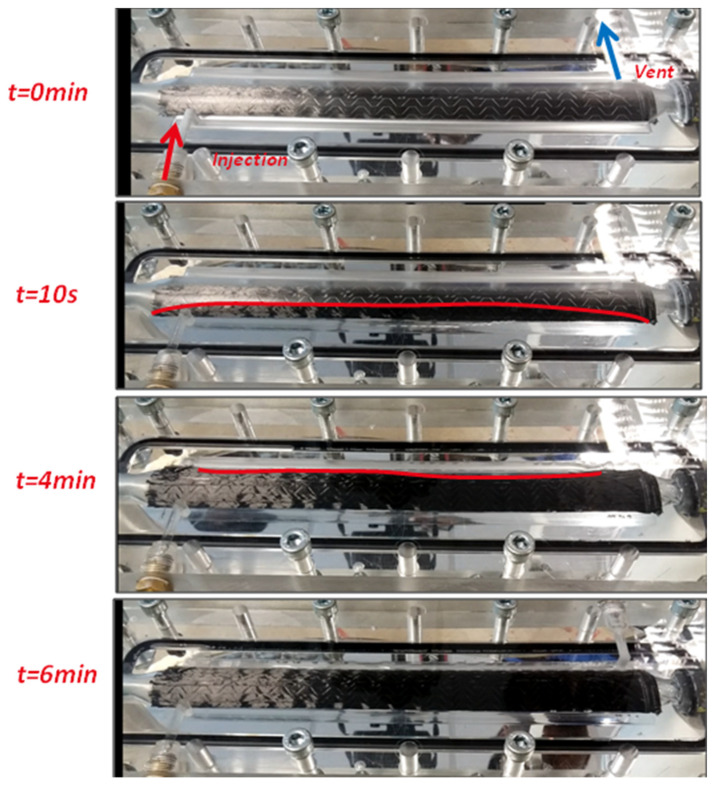
Flow front advancement for a tubular section using B-RTM process in an acrylic mold.

The initial trials of manufacturing Thick and Thin NCCF were carried out using the acrylic mold to check the draping and the impregnation behavior of the fabric preform and the injection strategy with TP and TS matrices. The advancement of the flow front was observed using a high-speed camera, which was set up perpendicularly to the transparent acrylic mold. An example of the above is shown in Figure 10, where the Thin NCCF preform was injected with epoxy matrix (Epolam 5015/5015).

Figure 10 shows the injection flow fronts at time t = 0 s, 10 s, 4 min, and 6 min. There were no anomalies observed, and the complete impregnation was achieved with the injection pressure of 3.8 bar and bladder pressure of 4 bar.

**Figure 11:**
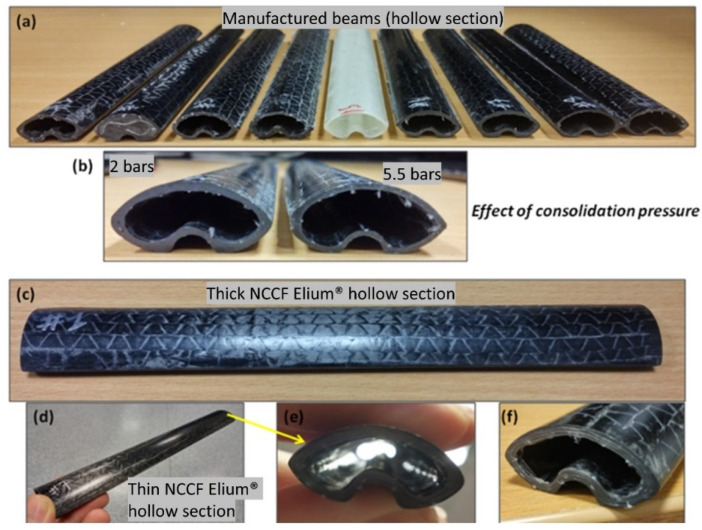
Manufactured Thin and Thick NCCF Elium^®^ Composite tubular sections (beams) and effect of consolidation pressure (**a**) different beams (**b**) effect of consolidation pressure (**c**) thick NCCF Elium hollow section (**d**) Thin NCCF Elium hollow section (**e**,**f**) cross section of thin Elium tube.

**Figure 12:**
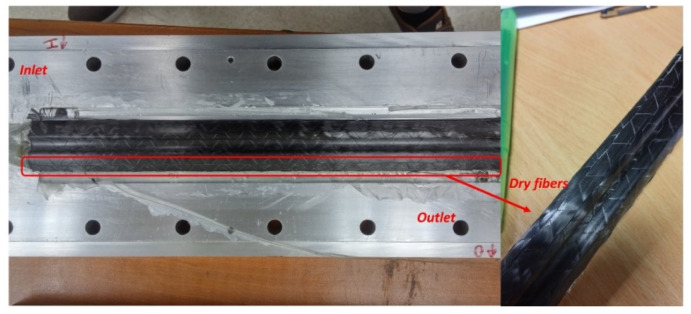
Cured part with process parameters (injection pressure 2 bar and bladder pressure 4 bar) showing unfilled preform at the outlet after the injection time of 22 min.

It should be noted that the whole study first started with a plain, glass weave followed by Thick and Thin NCCFs. As inferred from literature, it was essentially required that the bladder pressure should be higher than the resin injection pressure; otherwise, there are high chances that the bladder will collapse [9]. Hence, for all the trials, the bladder pressure was kept higher than the resin injection pressure. At this stage of the study, the major idea was to check the injection strategy, mold design used, and preform drapability. The consolidation of bladder pressure was also varied, depending on the injection and bladder pressure used. In total, 32 tubular beam sections were manufactured during the trial stage, and some of them are shown in Figure 11. Figure 11a shows the different manufactured hollow tubes, while Figure 11b shows the differences in the tube cross-sections with different consolidation pressures. Figure 11c–f shows the different views of tube and its cross-section depicting the uniform cross section and excellent surface finish. Additionally, the effect of bladder consolidation pressure can be seen with two different tubular sections consolidated at 2 bar and 5.5 bar, respectively (refer to Figure 11b). The target tubular section thickness was 1.5 mm and the ones achieved were 1.9 mm with 2 bar and 1.52 mm with 5.5 bar, respectively. The tubular section that was consolidated at 5.5 bar had 57% fiber volume fraction (V_f_) compared to 51% of the one that was consolidated at 2 bar. It should be noted that the conventional B-RTM approach for manufacturing composite tubes with high-permeability fibers with a pressure difference of 2 bars was found not suitable for injecting low-permeability thin plies and the preform was not fully filled at the outlet even with the injection time of 22 min (Refer Figure 12).

### 3.2. Process Parameters’ Optimisation

Once the mold design and the injection strategy were checked to be working fine, the next major step was to identify the fixed and variable process parameters and optimize the variables. The identified fixed and variable process parameters are depicted in Table 2. The fixed parameters included the FOE-sized, Thin NCCF Fabrics (0/45) for the whole optimization study, and Elium^®^ 150 as a TP resin with a fixed 2.5% benzoyl peroxide (BPO) as an initiator. Additionally, as the resin was cured at room temperature, the mold was kept at an ambient temperature. Furthermore, the post-fill bladder pressure was fixed at 5.5 bar for consolidation purpose, while the post-fill inlet boundary condition (BC) was clamped.

The important variable parameters, which were required to be optimized, were identified as filling resin pressure and filling bladder pressure, as recommended by various researchers [9,32,33]. Along with these major parameters, post-fill resin pressure, as well as filling and post-filling outlet boundary conditions, were required to be optimized. These parameters will not affect the impregnation time, but the quality of the tubular sections can be affected using different boundary conditions. The planned optimization cycle for producing high-quality and faster injection tubular sections is depicted in Table 3. The “tests” are referred as the specific condition at which the manufacturing was carried out.

Several test plans, shown in Table 3, served to compare the various process parameter optimization schemes to finally decide the optimal strategy to inject a full tubular section. In this study, 49 tubular sections were manufactured using different injection, bladder, and consolidation pressures and with different clamping strategies. Below, sub-sections will give more insight into the various test cases. Out of 49 tubular sections manufactured, seven of them represented one of the particular test cases, as discussed in Table 3. The manufactured tubes were reproducible in terms of final thickness (1.5 ± 0.05 mm) and the fiber volume fractions (e.g., 54 ± 0.8%) for a particular experiment repeated with the same boundary conditions.

#### 3.2.1. Process Parameters during Injection

**Figure 13:**
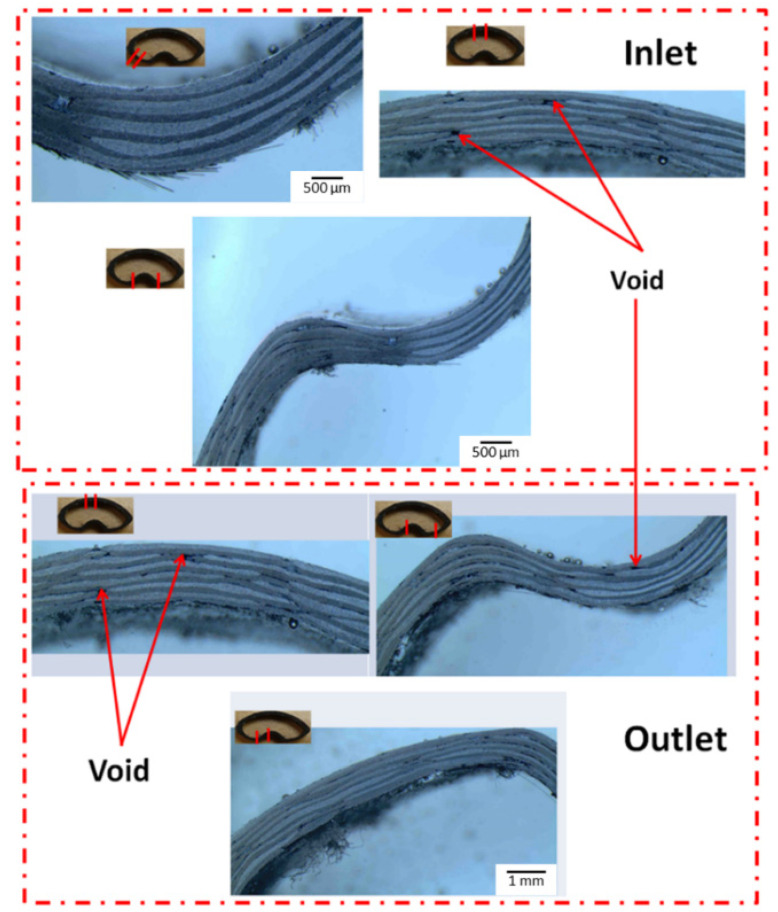
Microscopic images of the tubular section (Test 6) showing minimal void sites at multiple locations of the inlet and outlet sections.

In the first attempt, process parameters during injection were optimized. Table 4 shows the details of all the variants of the optimization trials. It should be noted that for each test at least three beams were manufactured to check the accuracy of the particular test case. Test 6 and Test 2 showed the two comparison injection schemes. For Test 6, the bladder pressure > resin injection pressure, while, for Test 2, the injection and bladder pressure were kept the same. An important observation was that the tubular section injected with the same bladder and resin pressure was injecting faster primarily because of less resistance to the resin flow due to comparable bladder pressures, whereas the injection time was 1 min longer in the case of the tubular section manufactured with a higher bladder pressure. The quality of manufactured tubular sections was investigated by both microscopy and ASTM D792/D2734 and found to be very similar for both injection schemes, although a slightly higher volume fraction was attributed to better consolidation in the case of Test 2. The tubular section was cut using a water jet diamond cutter at both the inlet and the vent positions. At least three samples were tested for both the inlet and outlet side to quantify the void content. The standard void tests were conducted on the manufactured tube following ASTM D792 [34] and ASTM D2734-09 [35]. The microscopy images of the Test 6 tubular section can be seen in Figure 13. Void content was calculated using a digital microscopy technique using an Olympus SZX7 to cover the full cross section of the tubular section. The images were captured using a digital camera and the mosaic images were constructed by joining the images obtained using the microscope. This technique ensured a complete observation of the sample showing the distribution of voids [36].

#### 3.2.2. Effect of Consolidation Pressure

**Figure 14:**
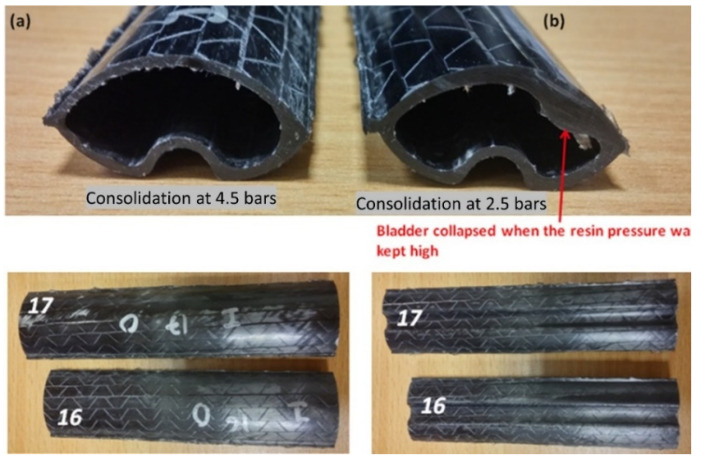
Effect of consolidation pressure on tubular sections manufactured using B-RTM process (**a**) consolidation at 4.5 bar (**b**) consolidation at 2.5 bar.

Another important aspect of the B-RTM process is to check the effect of consolidation pressure during manufacturing [9,32,33]. To check the effect, the results of the tubular section manufactured using Test 4 and Test 5 parameters were compared. As can be seen from Table 4, during Test 5, after the injection was completed, the outlet was clamped, and the pressure was still building in at 3 bar. Therefore, the consolidation pressure was 2.5 bar (Bladder pressure–Resin pressure) as opposed to 4.5 bar in Test 4 where the inlet was clamped, and the bladder pressure was subsequently increased to 5.5 bar. Test 5 with 2.5 bar consolidation pressure yielded a very low fiber volume fraction of 49% compared to 4.5 bar of consolidation pressure where 59% V_f_ was achieved. Although a lower injection time was 2.5 times faster, injection was achieved, which is desirable for mass production of composite tubular sections. Still, there was a huge sacrifice in terms of fiber volume fraction, which may affect the mechanical performance. In our recent research, we showcased the effect of the fiber volume fraction, fiber architectures, and the resin systems on the mechanical and vibration damping properties of the tubular composite shafts [13,14,15,37]. Additionally, it was noticed that when the resin pressure was kept high, there were instances of the bladder collapsing, as shown in Figure 14.

#### 3.2.3. Effect of Outlet Boundary Conditions (BC)

In the majority of the RTM process, a vacuum was used as an aid in removing any entrapped air from the fabric preform during the preform compaction prior to the injection and during the complete curing cycle as well as the consolidation phase. Herein, Test 1 was carried out with the bladder and resin pressure at 4 bar during the injection phase. The inlet was clamped at 11 min, and the bladder pressure was increased to 5.5 bar. However, at the outlet, a 500-mbar vacuum was kept throughout the curing cycle. However, in the case of Test 2, the outlet was left at atmospheric pressure. The results, as shown in Table 4, conveyed that there was no significant effect of outlet boundary conditions during the B-RTM process. The void contents measured using both techniques were similar for both the testing conditions, although it was noticed that the tubular sections with the vacuum as the outlet BC had lesser surface porosities.

#### 3.2.4. Effect of Matrix Systems

Although the main idea was to carry out the entire study with TP Elium^®^ resin, a few tubular sections were also manufactured with TS epoxy Epolam 5015/5015 resin for baseline comparison. Test 6, which was carried out with Elium^®^ resin, was repeated with the same process parameters but with TS epoxy resin. The NCCF epoxy tubular section took a slightly longer injection time than the NCCF Elium^®^ tubular section due to the lower viscosity in the case of the Elium^®^ 150 resin (100 cP) compared to 210 cP in the case of the Epolam 5015 resin. Additionally, the fiber volume fraction achieved with epoxy resin was higher due to the longer curing time of 24 h compared to 1.5 h in the case of Elium^®^ resin. The excess resin was squeezed out of the laminate, as the epoxy resin had longer gel time resulted and, hence, the laminates had comparatively higher V_f_.

### 3.3. Challenges and Solutions

**Figure 15:**
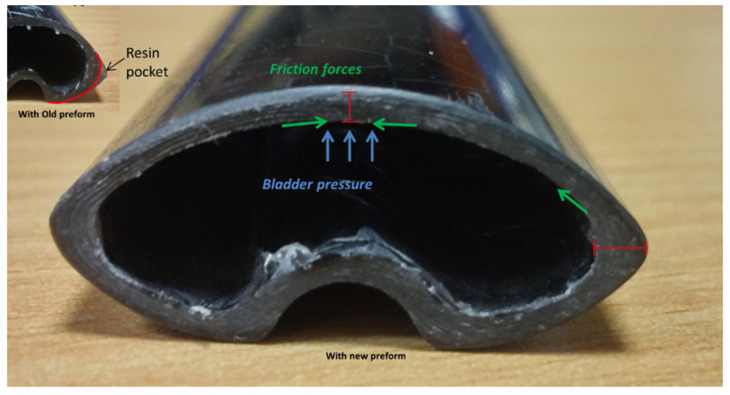
Tubular section showing better-filled corners with a larger preform.

Tubular sections were successfully manufactured using the above-discussed parameters with Thin NCCF (0/45) fibers as reinforcement and Elium^®^ and epoxy as the matrices. However, it was noticed that there was still a huge room for improvement to fasten the injection process and to eliminate the resin pockets, which were observed in many trials during the optimization process. It was decided to use a larger preform (370 mm × 250 mm) compared to the older preforms of 330 mm × 250 mm dimensions used in the optimization study. With a larger preform, corners are seen to be better filled (refer to Figure 15) but still were slightly thicker than the top surface as the friction force with the bladder was higher in the corners (corner thickening effect), which is always the case with tubular geometries manufactured with concave tools [38]. The sections that were manufactured with some collapsed bladder conditions tended to have more resin at the corners as well as at the collapsed region of the tube in the vicinity of the bladder collapse point. The thickness of the tube was found to be up to 2.1 mm as opposed to 1.5 mm due to a significant chunk of resin formation at the corners. To quicken the injection process, both the resin and bladder pressures during the injection were reduced with an aim to reduce the compactness of the fabric preform, which lowers the permeability of the fabric and, in turn, makes the injection longer. As seen from Table 5, Test 8 and Test 9 were repeated thrice with both the resin systems (TP and TS). During Test 8, higher resin injection and bladder pressures compared to Test 9 were used.

Lower injection and bladder pressures quickened the injection process as the preform was less compacted. Bladder pressure significantly influenced the compaction of the fabric preform. With the higher bladder pressure, the layers of the preform were pressed, and the permeability of the compressed preform was reduced and affected the fiber impregnation time. During the draping, the preform touched the plane surface of the mold first. Surface pressure was created along with the frictional force, which existed due to the friction between the layers of the tubular section preform and the mold cavity surface. Preferably, this frictional force should be lower than the tensile forces, which help in the movement of the preform into the edges of the mold cavity. If not, the drapability would be poorer with surface pressure preventing the draping into the edges [39]. There was lower permeability on the surface than the edges due to the strong fiber compaction at the planar surfaces. Hence, the choice of the bladder as well as reducing the bladder and resin pressure was significant for the desired flow front advancement.

**Figure 16:**
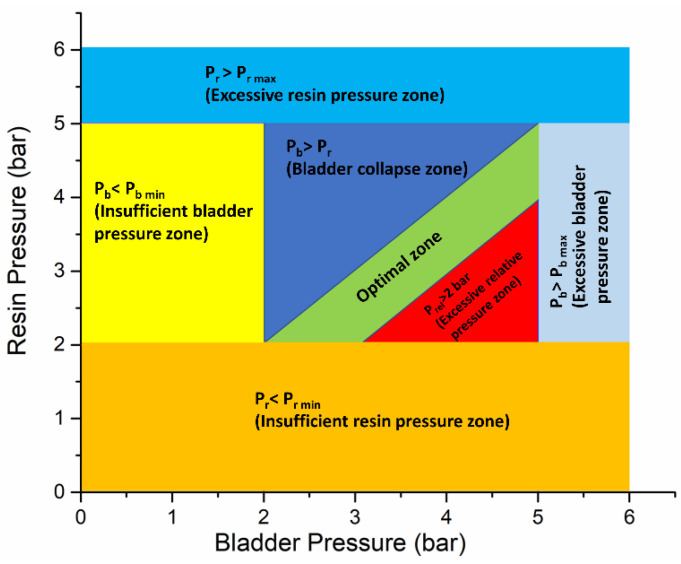
Manufacturing optimization zone diagram at different pressure boundary conditions during B-RTM process.

Based on the detailed experimental investigation at different boundary conditions, a moldability zone diagram was constructed, as shown in Figure 16, showing an optimal zone for effectively processing Elium- and Epoxy-based composite tubes with a B-RTM process. Different zones with their boundary conditions, as shown in Figure 16, are explained below.

1. Insufficient resin pressure zone (Resin Pressure P_r_ < Minimum Resin Pressure P_r min_): Minimum of 2-bar resin pressure was required to impregnate the preform with low-permeability, thin-ply carbon fabrics. Usage of P_r_ < 2 bar resulted in unfilled parts and a significantly higher filling time.
2. Insufficient bladder pressure zone (Bladder Pressure P_b_ < Minimum bladder Pressure P_b min_): The minimum bladder pressure required to fully inflate the nylon bladder was 2 bar below which the preform was not fully compacted, there was race tracking of the resin, and the parts remained unfilled. It should be noted that the minimum bladder pressure is dependent on the part geometry, which held true for the current investigation.
3. Excessive bladder pressure zone (Bladder Pressure P_b_ >Maximum bladder Pressure P_b max_): This zone should be avoided as the bladder pressure above 5 bar led to excessive compaction of the preform and was undesirable, especially while using the low-permeability, thin-ply preforms.
4. Excessive resin pressure zone (Resin Pressure P_r_ > Maximum Resin Pressure P_r min_): Excessively higher resin pressure is to be avoided to minimize the chances of race tracking and wrinkling of the fabric preform.
5. Bladder collapse zone (Bladder Pressure P_b_ > Resin Pressure P_r_): The bladder should not be kept higher than the resin pressure to avoid the collapsing of the preform during the injection.
6. Excessive relative pressure zone: The difference between bladder and resin pressures should be kept smaller. The parts injected at a higher pressure difference will significantly increase the injection time.

**Figure 17:**
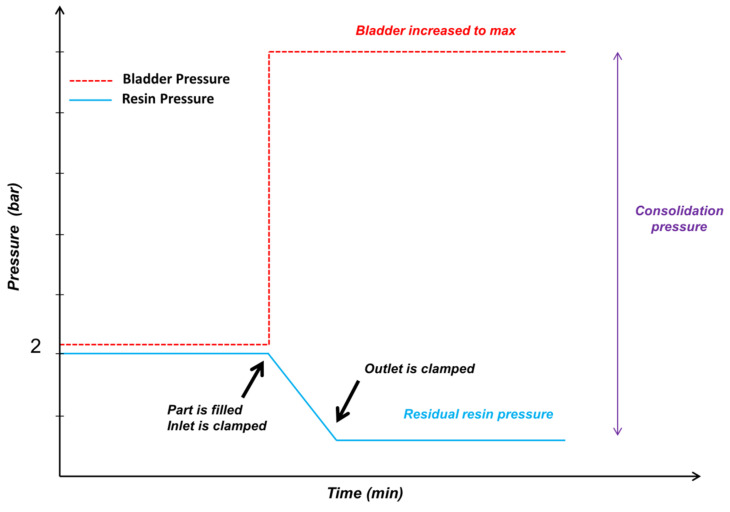
Deduced scheme for injecting a thin-ply tubular section with optimized parameters and boundary conditions.

The optimization process for the tubular section deduced the following findings: Bladder pressure determines the preform compaction. It would be easy to impregnate the fabrics at a lower bladder pressure, as that will cause less compaction of the fabric. However, at a lower bladder pressure it should be noted that, if resin pressure is higher than bladder pressure, there are chances of a bladder collapsing. Based on this optimization, the concluded scheme for injection of a tubular section is as shown below (Refer to Figure 17).

1. Throughout the injection phase, bladder pressure (2.2 bar) was kept higher than the injection pressure (2 bar).
2. When the part was filled, then the inlet was clamped and, simultaneously, the bladder was increased to a maximum of 5.5 bar.
3. Clamp the outlet after step 2, such that maximum consolidation pressure is achieved.

## 4. Conclusions

The B-RTM process was used to successfully manufacture a complex tubular section with thin-ply NCFs as the reinforcement material and thermoplastic Elium resin as the matrix. A detailed case study was presented, showing the specifics regarding the effective mold design, recommended process parameters, associated challenges, and the proposed solutions. Following are the salient finding from the research case study.

1. An injection scheme was deduced to inject successfully the Thin NCCF Elium^®^ and Epoxy composite sections. During the injection phase, bladder pressure (2.2 bar) should be kept higher than the resin injection pressure (2 bar). Once the part is filled, then the inlet should be clamped and, simultaneously, the bladder should be increased to a maximum of 5.5 to 6 bar or higher, depending on the final fiber volume fraction requirement. Further, the outlet should be clamped to achieve maximum consolidation pressure.
2. Using the optimized B-RTM process parameters, the tubular composite sections with minimal void content (<1%) and higher fiber volume fraction (>55%) can be manufactured using fibers with lower permeability and with thermoset and thermoplastic matrices of viscosity ≤ 250 cP.
3. Higher consolidation pressure is key to achieve an optimal fiber volume fraction, although there will be some sacrifice in terms of the injection speed. When the resin pressure was kept high at ≥ 3.8 bar, there were instances of the bladder collapsing.
4. There was no significant effect of outlet boundary conditions (500-mbar vacuum at an outlet or atmospheric pressure) during the B-RTM process. Both conditions had similar fiber volume fractions and void content, barring lesser surface porosities in the case of the former.

## Figures and Tables

**Table 1:** Permeability values for thick and thin NCCFs.

Fabric Type	K_x_ (m^2^)	K_y_ (m^2^)	K_z_ (m^2^)
Thin NCCFs (200 g/m^2^)	1.27 × 10^−11^	1.1 × 10^−11^	2.1 × 10^−13^
Thin NCCFs (400 g/m^2^)	2.1 × 10^−9^	8.7 × 10^−10^	-

**Table 2:** Fixed and Variable injection and post-injection parameters for manufacturing a tubular section.

Fixed Injection and Post-Injection Parameters		Variable Injection and Post-Injection Parameters
Post-fill bladder pressure	5.5 bar	Filling resin pressure
Post-fill inlet Boundary condition (BC)	Clamp	Filling bladder pressure
Fabric sizing	Thermoplastic FOE	Post-fill resin pressure
Mold temperature	Ambient	Filling outlet BC
BPO (%)	2.5%	Post-filling outlet BC

**Table 3:** Test Plan for optimizing B-RTM parameters for a tubular section.

Test	1	2	3	4	5	6	7
Bladder pressure during filling (bar)	4	4	3.8	3.8	3.8	4.2	4
Resin pressure during filling (bar)	4	4	3.8	4	4	4	3.8
Resin pressure post filling (bar)	500 mbar vacuum	Atm pressure	3	0	3	0	3

**Table 4:** Results from the process parameter optimization for various test cases for manufacturing a tubular section.

		Test 1	Test 2	Test 3	Test 4	Test 5	Test 6	Test 6	Test 7
Fibers		Thin NCCF 100/100 g/m^2^ (Elium^®^ sized)						Thin NCCF 100/100 g/m^2^ (Epoxy sized)	Thin NCCF 100/100 g/m^2^ (Elium^®^ sized)
Resin		Elium^®^ 150						Epolam 5015/5015	Elium^®^ 150
Bladder pressure (bar)		4	4	3.8	3.8	3.8	4.2	4.2	4
Resin pressure (bar)		4	4	3.8	4	4	4	4	3.8
Bladder pressure post-fill (bar)		5.5	5.5	5.5	5.5	5.5	5.5	5.5	5.5
V_f_ (%)		57 ± 0.22	55 ± 0.35	57 ± 0.34	59 ± 0.72	49.2 ± 0.31	57 ± 0.21	62 ± 0.27	54 ± 0.21
Injection time		5 min 35 s ± 21 s	3 min 40 s ± 11 s	4 min 20 s ± 14 s	2 min 30 s ± 8 s	50 s ± 3 s	4 min 4 s ± 12 s	6 min 30 s ± 12 s	6 min 30 s ± 10 s
Clamping and consolidation strategy		Clamp inlet at 3 min and increase bladder pressure to 5.5 bar.	Clamp inlet at 6 min 30 s and increase bladder pressure to 5.5 bar	Clamp the outlet and let the pressure build in with resin pressure at 3 bar. Increase bladder pressure to 5.5 bar	Clamp inlet at 3 min and increase bladder pressure to 5.5 bar	Clamp the outlet and let the pressure build in with resin pressure at 3 bar. Increase bladder pressure to 5.5 bar	Clamp inlet at 8 min and increase bladder pressure to 5.5 bar	Clamp inlet at 10 min and increase bladder pressure to 5.5 bar	Clamped outlet and let the pressure build in with resin pressure at 3 bar. Increase bladder pressure to 5.5 bar
Void Content (%) (Microscopy)	Inlet	<3	<2	<2	<2	<2	<2	<3	<2
	Outlet	<2	<2	<2	<2	<2	<1	<2	<2
Void Content (%) (ASTM D792/D2734)	Inlet	2.31 ± 0.53	1.14 ± 0.13	1.34 ± 0.19	1.19 ± 0.08	1.25 ± 0.13	0.97 ± 0.11	2.31 ± 0.45	1.09 ± 0.11
	Outlet	1.63 ± 0.27	1.31 ± 0.09	1.54 ± 0.30	1.07 ± 0.12	0.93 ± 0.11	1.04 ± 0.14	1.41 ± 0.16	1.41 ± 0.11

**Table 5:** Injection results during B-RTM process with reduced bladder and injection pressures.

	Test 8	Test 8	Test 9	Test 9
Fibers	Thin NCCF Epoxy sized	Thin NCCF Elium^®^ sized	Thin NCCF Epoxy sized	Thin NCCF Elium^®^ sized
Resin	Epoxy	Elium^®^ 150	Epoxy	Elium^®^ 150
Bladder pressure (bar)	4.2	4.2	2.2	2.2
Resin pressure (bar)	4	4	2	2
Bladder pressure post-fill (bar)	5.5	5.5	5.5	5.5
V_f_ (%)	53	52	56	55
Injection time	4 min	2 min 29 s	1 min 5 s	51 s
Clamping strategy	Clamp inlet at 5 min and increase bladder pressure to 5.5 bar.	Clamp inlet at 4 min and increase bladder pressure to 5.5 bar.	Clamp inlet at 2 min and increase bladder pressure to 5.5 bar.	Clamp inlet at 2 min and increase bladder pressure to 5.5 bar.
	Clamp outlet at 7 min	Clamp outlet at 5 min 30 s	Clamp outlet at 5 min 30 s	Clamp outlet at 4 min 30 s
	Outlet kept at vacuum 500 mbar	Outlet kept at vacuum 500 mbar	Outlet kept at vacuum 500 mbar	Outlet kept at vacuum 500 mbar

## Data Availability

The data presented in this study are available on request from the corresponding author.
